# New Appliances and Things Medical

**Published:** 1908-07-04

**Authors:** 


### NEW APPLIANCES AND THINGS MEDICAL.

[We shall be glad to receive at our Office, 28 & 29 Southampton Street, Strand, London, W.C., from the manufacturers, specimens of all new preparations and applianceswhich may be brought out from time to time.]

### CHEMICAL FIRE EXTINGUISHERS.

The British Fire Prevention Committee recently tested a new chemical fire extinguisher of the type known to most medical superintendents, but having some improvements of interest. The apparatus consists of the usual cylindrical vessel, about 24 in. in height and about 7 in. diameter, weighing when charged approximately 30 lbs., and containing 3 gallons of soda solution and an acid bottle. The principle is the usual one adopted in this class of extinguisher, *i.e.*, when the acid is mixed with the solution a considerable internal pressure is developed owing to the generation of gas, and the contents are squirted on the seat of the fire, which is extinguished by carbonic acid gas, then produced. The amount of liquid thrown, it will be noticed, is small, but the effect may be roughly stated as greater than that of 100 gallons of water. The body of the apparatus is made of copper, tinned inside, the vertical seam being strongly rivetted and the top brazed. The acid bottle is made of glass, and is held in a cage at the top of the vessel; it is closed at the top end with a lead stopper, which, while making a fair joint whilst in position, is quite free to drop out upon the extinguisher being turned upside down. The acid then mingles with the solution, and the action described takes place. Normally the hose hangs down from the top, thus being free from kinks or bends, a position which enhances its chance of durability, whilst in the working position it is best situated for convenient use, and when out of use is not subjected to the deteriorating influence of the acid or the solution. In these respects the apparatus is similar to the best forms of other extinguishers of this kind, but the principal improvement lies in the position of the acid bottle and the method of ensuring its contents mixing with the liquid solution when the extinguisher is inverted for use. There is an almost similar apparatus, made by a well-known firm, wherein, upon the extinguisher being inverted, the acid bottle falls on to a spiked projection at the end of the outer vessel, and is thus broken if the acid bottle is made of glass, or the lead capsule pierced if a lead bottle is used. The bottle travels between guides provided for the purpose, but in the case of the present improved extinguisher, the guides are dispensed with, the bottle is fixed, and there is no need either to break it or to pierce the stopper, which simply falls out, being quite loose.

This is the first chemical extinguisher tested by the Fire Prevention Committee, and the report was to the effect that it is generally efficient as a "first-aid" apparatus, for which purpose it is intended. Chemical fire extinguishers frequently, while putting out an actual fire in substances such as wool, will not altogether prevent subsequent smouldering, but this can easily be dealt with when the fire is subdued. On the other hand, they will subdue naphtha, celluloid, tar, and petroleum when fired, and other inflammable substances for which water would be practically useless.

Probably there are few institutions unprovided with some make of chemical extinguisher, but unfortunately it is not usually deemed necessary to have "fire drills" with them as with the usual water fire appliances. This is a mistake. The staff or inmates should be taught drill with them simply for the purpose of ensuring familiarity in handling the extinguishers, just as they would drill with a hose or fire engine. This particular extinguisher is called the "Accurate," and has been supplied to the Charing Cross Hospital. It is also used by the London General Omnibus Company on their motor omnibuses, the Standard Oil Company and others, and is sold by the Underwriters Fire Appliances, Limited, 30 Fleet Street, London, E.C., who will give private demonstrations by request.

